# Facile Synthesis of Sponge-Like Porous Nano Carbon-Coated Silicon Anode with Tunable Pore Structure for High-Stability Lithium-Ion Batteries

**DOI:** 10.3390/molecules26113211

**Published:** 2021-05-27

**Authors:** Shugui Song, Jingcang Li, Anqi Zheng, Yongqiang Yang, Kuibo Yin

**Affiliations:** 1 SEU-FEI Nano-Pico Center, Key Laboratory of MEMS of Ministry of Education, Southeast University, Nanjing 210096, China; songshugui@seu.edu.cn (S.S.); 220191413@seu.edu.cn (J.L.); zhengaq@seu.edu.cn (A.Z.); 2 National Graphene Products Quality Supervision and Inspection Center (Jiangsu), Jiangsu Province Special Equipment Safety Supervision Inspection Institute, Wuxi 214174, China

**Keywords:** carbon-coated silicon, porous structure, mechanical simulation, lithium-ion battery

## Abstract

To address the challenge of the huge volume expansion of silicon anode, carbon-coated silicon has been developed as an effective design strategy due to the improved conductivity and stable electrochemical interface. However, although carbon-coated silicon anodes exhibit improved cycling stability, the complex synthesis methods and uncontrollable structure adjustment still make the carbon-coated silicon anodes hard to popularize in practical application. Herein, we propose a facile method to fabricate sponge-like porous nano carbon-coated silicon (sCCSi) with a tunable pore structure. Through the strategy of adding water into precursor solution combined with a slow heating rate of pre-oxidation, a sponge-like porous structure can be formed. Furthermore, the porous structure can be controlled through stirring temperature and oscillation methods. Owing to the inherent material properties and the sponge-like porous structure, sCCSi shows high conductivity, high specific surface area, and stable chemical bonding. As a result, the sCCSi with normal and excessive silicon-to-carbon ratios all exhibit excellent cycling stability, with 70.6% and 70.2% capacity retentions after 300 cycles at 500 mA g^−1^, respectively. Furthermore, the enhanced buffering effect on pressure between silicon nanoparticles and carbon material due to the sponge-like porous structure in sCCSi is further revealed through mechanical simulation. Considering the facile synthesis method, flexible regulation of porous structure, and high cycling stability, the design of the sCCSi paves a way for the synthesis of high-stability carbon-coated silicon anodes.

## 1. Introduction

With the rapid growth of energy demand in mobile devices and electric vehicles, etc., the development of lithium-ion batteries with a high capacity, long cycle life, and low cost has been paid much more attention around the world [1]. Silicon has become one of the candidates with the most potential to replace commercial graphite anode (theoretical specific capacity is only 372 mAh^−1^ for LiC_6_) due to the high theoretical specific capacity of 4200 mAh g^−1^ for Li_22_Si_5_ [2,3,4]. However, the extremely large volume expansion of silicon anode as high as 280% (vs. Li_15_Si_4_)~417% (vs. Li_22_Si_5_) causes severe problems such as pulverization, unstable solid electrolyte interface (SEI) film, etc. [5,6]. These issues lead to the rapid irreversible capacity decay of silicon anodes during the cycling, and then result in poor cycling stability.

Structure design is one of the main strategies to improve the cycling stability of silicon-based anodes. Carbon-coated silicon’s design exhibits improved structural stability and higher cycling stability due to the protection of silicon by the carbon coating layer [7,8,9]. For example, Liu et al. [10] proposed a hierarchical structure design inspired by the structure of pomegranate through a evaporation-driven self-assembly method combined with coating and etching processes. In this structure, the silicon nanoparticles are encapsulated by a conductive carbon layer that leaves enough room for expansion during lithiation. Owing to the more stable SEI film and relieved stress, this pomegranate-like silicon-based anode exhibited a superior cyclability (97% capacity retention after 1000 cycles). Ryu et.al. [11] introduced a two-dimensional silicon nanosheet coated with a thin carbon layer through chemical vapor deposition. This carbon-coated silicon nanosheet formed ripples upon delithiation, which effectively releases the induced stress, rendering the electrode much more stable and durable than the uncoated counterparts. However, although these improved carbon-coated silicon designs show excellent cycling stability, the complex synthesis methods and even uncontrollable structure adjustment still limit the practical application of carbon-coated silicon anodes.

Herein, we design a sponge-like porous nano carbon-coated silicon (sCCSi) anode with a tunable pore structure based on a facile synthesis method. It was found that the water adding strategy and the heating rate of pre-oxidation are the two key conditions for forming the sponge-like porous structure and maintaining the material integrity. Moreover, the sponge-like porous structure can be easily manipulated by changing the stirring temperature of precursor solution and the oscillation methods of precipitates. The sCCSi with normal and excessive silicon-to-carbon ratios, 1:5 and 1:2, both maintain high capacity retention up to 70.6% and 70.2% after 300 cycles at 500 mA g^−1^, respectively. Furthermore, the enhanced buffering effect on pressure brought from the sponge-like porous structure in sCCSi is revealed by mechanical simulation.

## 2. Results and Discussion

### 2.1. Synthesis and Characterization of sCCSi

**Figure 1:**
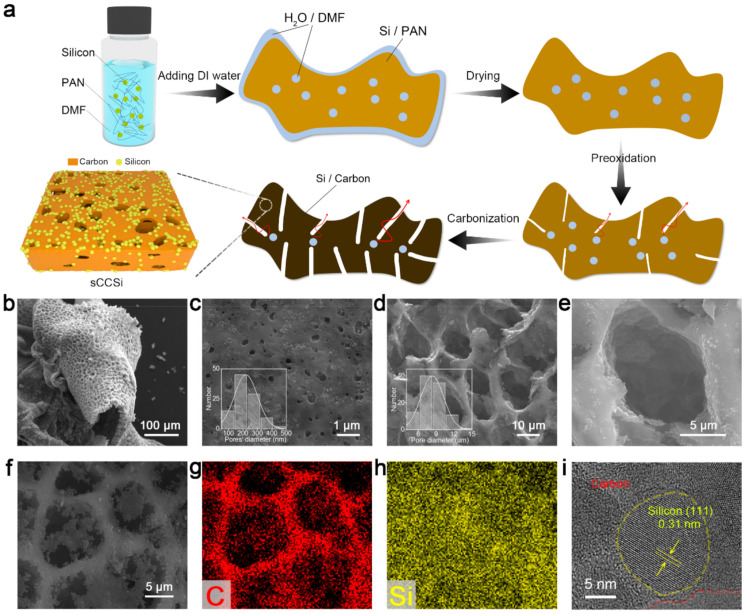
Synthesis process and structural characterization of sCCSi. (**a**) Schematic illustration for the synthesis process of sCCSi. SEM images of the (**b**) sponge-like porous structure, (**c**) small pores, and (**d**) large pores of sCCSi. The insets are the statistical distribution of corresponding pore diameters. (**e**) Enlarged view of the pore. SEM image of the (**f**) sponge-like skeleton of sCCSi, and the corresponding EDS mapping of (**g**) carbon element and (**h**) silicon element. (**i**) High-resolution TEM image of sCCSi.

The synthesis process of sCCSi is illustrated in Figure 1a. Silicon nanoparticles and polyacrylonitrile (PAN) are mixed in *N, N*-Dimethylacetamide (DMF) solution as precursor solution. De-ionized water (DI water) is then added to the precursor solution, which causes the exchange of two solutes (DMF and DI water). Afterwards, PAN is precipitated out due to the limited solubility in DI water [12,13,14]. At the same time, silicon nanoparticles are uniformly coated by PAN to form the PAN-coated silicon (Si/PAN) precursor. The process of precipitation is shown in Figure S1. Notably, the surface and interior of the Si/PAN precursor inevitably contain some water or DMF during the gradual precipitation process. In the following drying process, the water on the surface of the precursor evaporates to form a rough surface. In the further pre-oxidation process at 280 °C with a heating rate of 1 °C min^−1^, the high temperature is enough to evaporate the water or DMF inside the precursor, which will create many pores forming a sponge-like porous structure. Finally, sCCSi is obtained after carbonization. Compared with other methods for the synthesis of silicon-based anodes [8,15,16,17,18,19,20,21,22,23], our synthesis method is easier, as shown in Table S1. The structural characterization of sCCSi is shown in Figure 1b–i. Figure 1b clearly shows the sCCSi is with a sponge-like porous structure. Moreover, the pore diameter is observed over a large range from hundreds to thousands of nanometers. Two typical porous structures with small pores and large pores are shown in Figure 1c,d, and the mean diameters are 191 nm and 8.3 μm, respectively. The detailed statistical distribution and statistical data of the typical pore diameter are shown in the insets of Figure 1c,d and in Table S2. An enlarged view of a large sponge-like pore is shown in Figure 1e, where a rough inner wall is observed. The sponge-like carbon skeleton of sCCSi is further revealed from Figure 1f and its corresponding EDS mapping of carbon element (Figure 1g). In addition, the EDS mapping of the silicon element (Figure 1h) indicates that the silicon nanoparticles are relatively uniformly distributed. Furthermore, a typical coating microstructure is shown in the high-resolution TEM image (Figure 1i), in which the crystalline silicon nanoparticle with a lattice spacing of 0.31 nm is encapsulated by the amorphous carbon phase.

**Figure 2:**
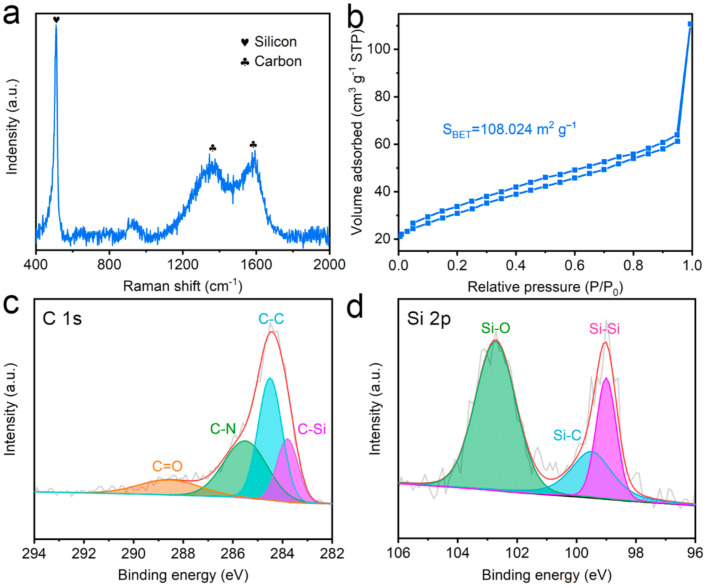
Structural and compositional characterization of sCCSi. (**a**) Raman spectrum of sCCSi. (**b**) Nitrogen isothermal adsorption–desorption curve of sCCSi. High-resolution X-ray photoelectron spectrum (XPS) spectrum of (**c**) C 1s and (**d**) Si 2p of sCCSi.

The structural and compositional characteristics of sCCSi are further studied. Figure 2a shows the Raman spectrum of sCCSi, in which the sharp peak at 506 cm^−1^ corresponds to crystalline silicon [24] and the two peaks at 1350 cm^−1^ and 1578 cm^−1^ correspond to the D and G bands of carbon material, respectively [8,25]. The intensity ratio of the D and G bands is 0.944 which indicates the carbon material has a few disordered structures with good conductivity as well [26,27]. The high conductivity is also confirmed by the electrochemical impedance spectroscopy, as shown in Figure S2, from which a low resistance of 61.68 Ω is obtained. To measure the specific surface area of sCCSi, a nitrogen isothermal adsorption–desorption test is performed, as shown in Figure 2b. Based on the Brunauer–Emmett–Teller (BET) model, the specific surface area of sCCSi is calculated to be 108.024 m^2^ g^−1^. The large specific surface area can be attributed to the sponge-like porous structure. Notably, the nitrogen adsorption–desorption curve is not completely closed in the low-pressure hysteresis loop which can be attributed to the filling or adsorption in micropores [28]. Furthermore, the chemical bonding states of sCCSi are further investigated through XPS. The full spectrum of sCCSi is shown in Figure S3, which indicates that the sample is composed of silicon and carbon. The high-resolution C 1s spectrum (Figure 2c) can be deconvoluted into four peaks (centered at 288.5, 285.5, 284.5, and 283.8 eV), which can be ascribed to C=O, C–N, C–C, and C–Si bonds, respectively [29,30] In addition, the high-resolution Si 2p spectrum (Figure 2d) can also be deconvoluted into three peaks, in which the peaks centered at 102.7, 99.5, 99.0 eV can be ascribed to Si–O, Si–C, and Si–Si bonds, respectively [30,31]. The presence of C–Si or Si–C bonds indicates a stable chemical bond is formed between silicon nanoparticles and carbon material.

### 2.2. Regulation of Sponge-Like Porous Structure

**Figure 3:**
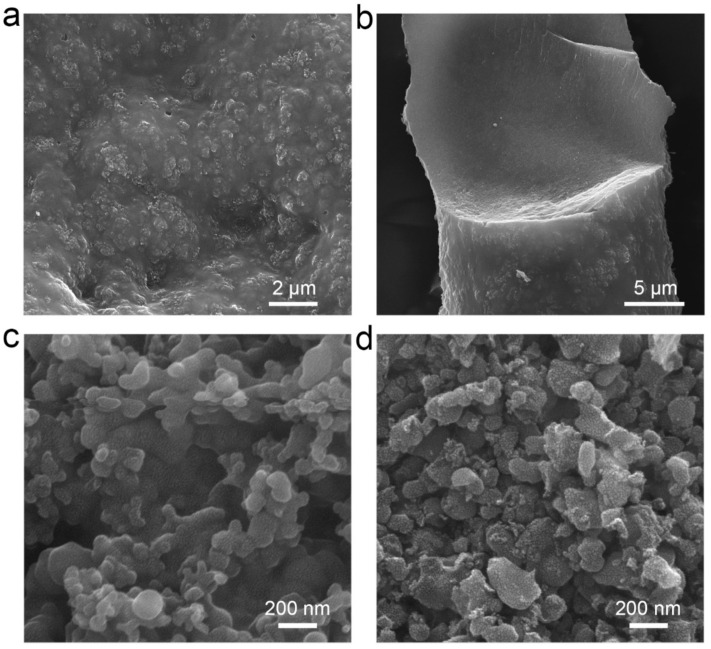
The impact of water adding strategies and heating rate of pre-oxidation on sponge-like porous structure. SEM images of (**a**) the morphology and (**b**) the cross-section of CCSi-w. SEM images of the morphologies of (**c**) CCSi-v and (**d**) CCSi-o.

The formation mechanism of the sponge-like porous structure was further explored. It was found that the sequence of addition of DI water plays an essential role in the precipitation of Si/PAN precursor during the synthesis process. Therefore, the impact of the opposite water adding strategy, namely adding the Si/PAN solution to DI water, on the sponge-like porous structure has been studied firstly. As shown in Figure 3a, the nano carbon-coated silicon composite prepared by the opposite adding strategy, denoted as CCSi-w, has a rough and compact surface. In addition, the cross-section image (Figure 3b) also shows that CCSi-w has a compact structure. It was thought that the different moisture content in the precipitate’s body caused by the different precipitation rates might be the possible reason for the compact structure [32], as shown in Figure S5a. Meanwhile, the relationship between the heating rate of pre-oxidation and the sponge-like porous structure was further studied. Two kinds of nano carbon-coated silicon composites were prepared through the different pre-oxidation drying methods (vacuum drying at 200 °C (CCSi-v) and oven drying at 200 °C (CCSi-o)). As shown in Figure 3c, no obvious pores exist in CCSi-v, but many relatively independent small particles can be seen on its surface, which can be attributed to the breakage and collapse of CCSi-v. The rapid heating rate (3 °C min^−1^ to 5 °C min^−1^) in the vacuum drying process will make the moisture or DMF in the Si/PAN precipitate evaporate faster, which might cause the collapse [33]. On the other hand, a more significant collapse is observed in CCSi-o with smaller particles on its surface, as shown in Figure 3d. It is consistent with the much higher heating rate (>5 °C min^−1^) in the oven drying process which might induce the increased crushing situation. The possible mechanism is shown in Figure S5b. Compared with the sCCSi prepared by the original method, the water adding strategy is found to be a key factor to form a sponge-like porous structure. Moreover, a slow heating rate in pre-oxidation process is found to be the key in keeping the porous morphology and preventing material from collapse.

**Figure 4:**
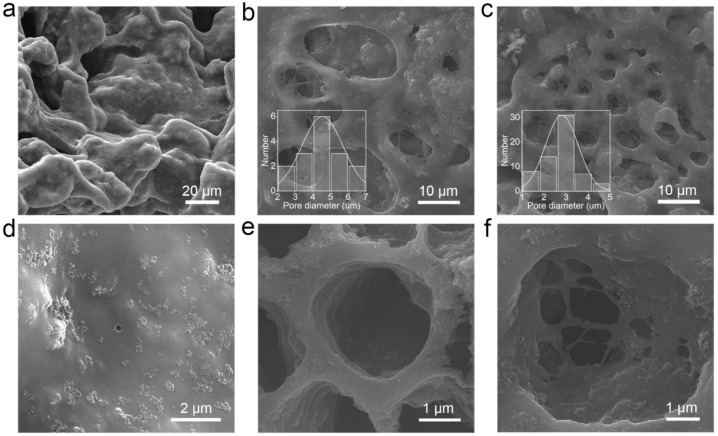
The impact of the stirring temperature and oscillation method on sponge-like porous structure. SEM images of the morphologies and structures of the CCSi obtained through three different processing methods: (**a**,**d**) CCSi-HT-U, (**b**,**e**) CCSi-HT-M, and (**c**,**f**) CCSi-RT-U. The insets in (**b**,**c**) are the statistical distribution of corresponding pore diameters.

The impacts of stirring temperatures of Si/PAN solution (high temperature and room temperature) and oscillation methods of Si/PAN precipitate (ultrasonic and mechanical oscillation) on the porous structure were also investigated. Three kinds of nano carbon-coated silicon composites are prepared: CCSi-HT-U, CCSi-HT-M, and CCSi-HT-U. In the synthesis process of CCSi-HT-U, the stirring temperature is set at 80 °C and the oscillation method is set to ultrasonic oscillation. In the synthesis process of CCSi-HT-M, the stirring temperature is set at 80 °C and the oscillation method is set to mechanical oscillation. In the synthesis process of CCSi-HT-U, the stirring temperature is set at room temperature and the oscillation method is set to ultrasonic oscillation. The corresponding material characterization results are shown in Figure 4 and Figure S4. Few pores can be observed in CCSi-HT-U, as shown in Figure 4a,d and Figure S4a. In contrast, there are relatively obvious pores on the surface of CCSi-HT-M, as shown in Figure 4b,e and Figure S4b. The mean pore diameter is calculated to be 4.5 μm. For CCSi-RT-U, the number of pores increases significantly, and the mean pore diameter decreases to 2.8 μm, as shown in Figure 4c,f and Figure S4c. The statistical distribution of the pore diameter of CCSi-HT-M and CCSi-RT-U are shown in the insets of Figure 4b,c, respectively. Through the comparison of the CCSi-HT-U, CCSi-HT-M, and CCSi-RT-U, the following conclusions can be drawn: (1) Stirring at room temperature tends to create more pores than stirring at high temperature. (2) Ultrasonic oscillation tends to produce smaller pores. More pores can be contributed to the solubility of PAN at different temperatures [34,35]. The possible reason for smaller pores is that ultrasonic oscillation makes the impact of water on the material more uniform and denser due to the smaller amplitude and higher frequency. The possible mechanisms of the stirring temperature and oscillation method are shown in Figure S5c,d, respectively.

### 2.3. Cycling Stability of sCCSi

**Figure 5:**
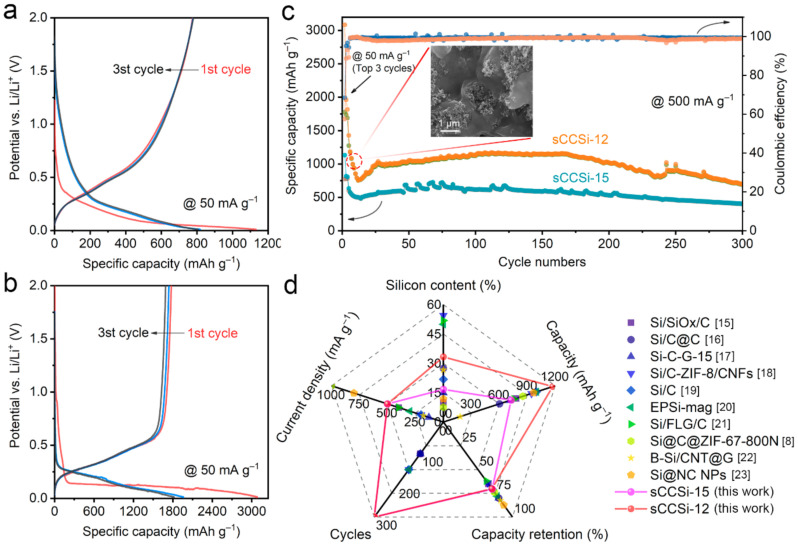
Electrochemical performance of sCCSi. Charging/discharging profiles of (**a**) sCCSi-15 and (**b**) sCCSi-12 at 50 mA g^−1^. (**c**) Cycling performance of sCCSi-15 and sCCSi-12 at 500 mA g^−1^, and the inset is the SEM image of sCCSi-12. (**d**) Comparison of sCCSi with reported silicon-based anodes [8,15,16,17,18,19,20,21,22,23] based on multiple dimensions.

To investigate the cycling stability of the sCCSi, the half-cells are assembled using two sCCSi materials with the normal and excessive silicon-to-carbon ratios of 1:5 and 1:2, which are denoted as sCCSi-15 and sCCSi-12, respectively. Figure 5a,b show the top three cycles’ charging/discharging profiles at 50 mA g^−1^ of sCCSi-15 and sCCSi-12, respectively. In the first cycle, sCCSi-15 and sCCSi-12 exhibit the capacities of 3084.1 mAh g^−1^ and 1131.9 mAh g^−1^, respectively. The larger specific capacity of sCCSi-12 can be attributed to the higher silicon-to-carbon ratio. However, the initial coulombic efficiency of sCCSi-15 (68.5%) is higher than that of sCCSi-12 (57.6%). Moreover, in the next two cycles, the coulombic efficiency of sCCSi-15 is about 96%, while that of sCCSi-12 is only about 90%. It can be attributed to the more exposed new surface of sCCSi-12 caused by the serious material fragmentation, which thus consumes more electrolyte to form SEI film [36,37]. The cycling stability of sCCSi is further revealed, as shown in Figure 5c. In the early stage of the cycling test, a rapid specific capacity decline of sCCSi-12 is observed. As shown in the inset in Figure 5c, the silicon nanoparticles fill the porous structure as well as the surface of sCCSi-12. This kind of structure can increase the shedding pulverization risk of sCCSi, which will cause rapid irreversible capacity loss. The specific capacities remained relatively stable after about 30 cycles. The highest discharging specific capacities of sCCSi-12 and sCCSi-15 are up to 1173.1 mAh g^−1^ and 726.7 mAh g^−1^ in the following cycles. Furthermore, sCCSi-15 shows an excellent capacity retention of 70.6% after 300 cycles. Moreover, sCCSi-12 also maintains excellent cycling stability, with 70.2% capacity retention after 300 cycles. Notably, the capacity fluctuation during cycling is related to the SEI film [38]. These results indicate sCCSi has excellent capacity stability, proving the validity of sponge-like porous structure design. Considering the facile synthesis process and flexible regulation of the porous structure, the excellent capacity stability of sCCSi is highly competitive with those reported for lithium-ion batteries [8,15,16,17,18,19,20,21,22,23], as shown in Figure 5d and Table S3.

**Figure 6:**
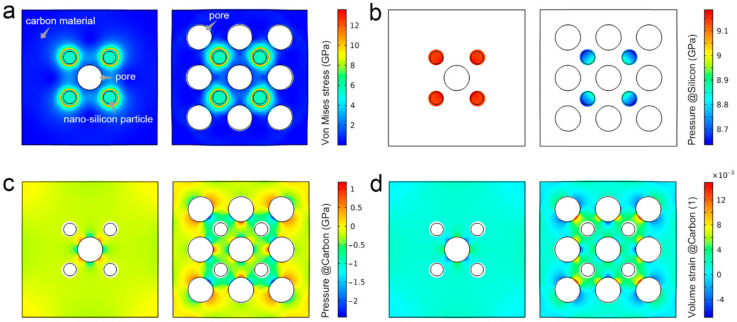
Mechanical mechanism of sponge-like porous structure on the structural stability of sCCSi. (**a**) The distribution of von Mises stress in CCSi and sCCSi models. (**b**) The distribution of pressure in silicon nanoparticles in CCSi and sCCSi models. (**c**) The distribution of pressure in carbon material in CCSi and sCCSi models. (**d**) The distribution of volume strain in carbon material in CCSi and sCCSi models.

To further reveal the mechanism of the sponge-like porous structure on the structural stability of sCCSi, the mechanical finite element simulation was performed. Two two-dimensional models of nano carbon-coated silicon (CCSi) and sCCSi were built for comparison, as shown in Figure 6a. They all contain four 25 nm silicon nanoparticles. The CCSi model only contains one 50 nm pore in the center used as a fixed boundary, while the sCCSi model contains nine 50 nm pores wherein the central pore is set to be a fixed boundary. According to the von Mises stress distribution in CCSi and sCCSi models (Figure 6a), the maximum stress is mainly concentrated at the contact interface between the carbon material and the silicon nanoparticles, with less stress in the silicon nanoparticles. It indicates that the carbon material has a positive buffering effect on silicon nanoparticles [39,40]. However, compared with the CCSi model, the pressure which represents the force per unit area on silicon nanoparticles caused by carbon material is significantly reduced, especially on the pores’ side, as shown in Figure 6b. Although the pressure in carbon material shows a slight increase (Figure 6c), it is acceptable compared with the greater pressure on the silicon nanoparticles. The less pressure on the silicon nanoparticles means improved structural stability, which can be attributed to the huge buffer space provided by the sponge-like porous structure [41]. It is further confirmed by the volume strain of CCSi and sCCSi models, as shown in Figure 6d and Figure S6, in which the volume strain of sCCSi is relatively large. Notably, the relief of pressure is more important than the slightly increased volume strain for nano carbon-coated silicon composite [11]. These results indicate the sCCSi not only has a protective effect from carbon material on silicon nanoparticles but also has an enhanced buffering effect on pressure between the silicon nanoparticles and carbon material due to the sponge-like porous structure.

## 3. Conclusions

In conclusion, we propose a facile method for the synthesis of sponge-like porous nano carbon-coated silicon (sCCSi) composite with tunable pores. The formation mechanisms of the sponge-like porous structure are revealed: (1) The strategy of adding water to the Si/PAN solution is an essential condition for the formation of the sponge-like porous structure. (2) A slow heating rate in the pre-oxidation process is the key to maintain material integrity. (3) The porous structure can be modified through the stirring temperature and oscillation methods. Specifically, stirring Si/PAN solution at room temperature combined with ultrasonic oscillation on Si/PAN precipitate tends to create more and smaller pores. Owing to the inherent material properties and sponge-like porous structure, sCCSi exhibits high conductivity, large specific surface area, and stable chemical bonding which are confirmed through Raman, nitrogen isothermal adsorption–desorption, and XPS analyses. As a result, sCCSi-15 and sCCsi-12 exhibit excellent capacity retentions up to 70.6% and 70.2% after 300 cycles at 500 mA g^−1^, respectively, which fully demonstrates the effectiveness of the sponge-like porous structure design in sCCSi. Furthermore, the enhanced buffering effect on the pressure between silicon nanoparticles and carbon material due to the sponge-like porous structure is revealed through mechanical simulation. These results pave the way for the design of high-stability carbon-coated silicon anodes through a facile and controllable method.

## 4. Experimental Methods

### 4.1. Synthesis of sCCSi

Silicon nanoparticles (99.9%, 30 nm, Macklin Inc., Shanghai, China) and polyacrylonitrile (PAN, *M*_w_ 150,000, Macklin Inc., Shanghai, China) were mixed in *N, N*-Dimethylformamide (DMF, analytically pure (99.8%), Sinopharm Chemical Reagent Co. Ltd., Shanghai, China) at a specific mass ratio, such as 1:5 or 1:2, and then stirred (400 rpm) at room temperature for 12 h to obtain a homogeneous Si/PAN precursor solution. Then, deionized water (DI water) was added dropwise to the Si/PAN precursor solution. PAN precipitated at the time, and the silicon nanoparticles that uniformly mixed with PAN were coated in its body to form a Si/PAN precipitate. The precipitate was then processed through mechanical oscillation for 10 min. Afterwards, the Si/PAN obtained was dried at 40 °C for 24 h, then pre-oxidized at 280 °C for 5 h with a heating rate of 1 °C min^−1^, and finally carbonized at 800 °C for 2 h with a heating rate of 5 °C min^−1^ to obtain the sponge-like porous nano carbon-coated silicon (sCCSi).

### 4.2. Synthesis of Nano Carbon-Coated Silicon Composites Used for Comparative Studies

The nano carbon-coated silicon composites used for comparative studies, such as CCSi-w, CCSi-v, CCSi-o, CCSi-HT-U, CCSi-HT-M, and CCSi-RT-U, were obtained by changing one or two methods at the same time in the original synthesis process. Specifically, CCSi-w was obtained by changing the order of adding water; namely, the mixed solution was added dropwise to DI water. CCSi-v and CCSi-o were obtained by setting the peroxidation method as vacuum drying and oven drying, respectively. CCSi-HT-M was obtained by setting the stirring temperature to 80 °C combined with the mechanical oscillation on Si/PAN precipitate. CCSi-RT-U and CCSi-HT-U were obtained by setting the stirring temperature to room temperature (RT, 25 °C) and 80 °C, respectively, combined with ultrasonic oscillation on Si/PAN precipitate. These materials and their corresponding synthesis methods are listed in Table S4.

### 4.3. Material Characterization

The morphologies and structure of the samples were observed using scanning electron microscopy (SEM, Inspect F50, FEI Company, Oregon, OR, USA) and transmission electron microscopy (TEM, Tecnai G2 20, Thermo Fisher Scientific Inc., Waltham, MA, USA). An energy-dispersive X-ray spectrum (EDS) instrument attached to the SEM apparatus was used for the elemental analysis of sCCSi. The crystal structures were characterized using the Raman spectrum (LabRAM HR UV-Visible, Horiba Jobin Yvon, Piscataway, NJ, USA). The BET specific surface area of sCCSi was measured by nitrogen isothermal adsorption–desorption test (Autosorb-IQ-XR, Anton Paar QuantaTec Inc., Boynton Beach, FL, USA). The interface contact of sCCSi was measured by X-ray photoelectron spectrum (XPS, ESCALAB 250Xi, Thermo Fisher Scientific Inc., Waltham, MA, USA).

### 4.4. Electrochemical Measurements

The electrode active material (sCCSi), polyacrylic acid (PAA, *M*_w_ 450,000, Aladdin Chemistry Co. Ltd., Shanghai, China) binder, and conductive agent (Super P) was mixed with the mass ratio of 8:1:1 and ground to use as the electrode slurry. Then, the slurry was coated on the copper foil and dried at 120 °C for 10 h in a vacuum environment to obtain the electrodes. The CR2032 type half-cells were assembled with the lithium sheet as the counter electrode and celgard 2400 as the separator. The electrolyte was 1 M LiPF_6_ in ethylene carbonate, diethyl carbonate, and dimethyl carbonate (1:1:1 *w*/*w*/*w*) mixture with 5 vol% fluoroethylene carbonate. The cells were aged for 24 h before the test and then placed on the LAND battery test system for galvanostatic charging/discharging test. The top three cycles were tested at the current density of 50 mA g^−1^ to activate the cells, and then the cycling test of 300 cycles was performed at a high current density of 500 mA g^−1^. Electrochemical impedance spectrum (EIS) was conducted on a Gamry Reference 3000 electrochemical workstation with a frequency range from 0.01 Hz to 1 MHz at 5 mV.

### 4.5. Mechanical Simulation

To reveal the mechanical mechanism of sponge-like porous structure on structural stability, two 270 nm × 270 nm two-dimensional models of CCSi and sCCSi were built. The CCSi model contained one 50 nm pore in the center and four 25 nm silicon particles. The sCCSi model contained nine 50 nm pores and four 25 nm silicon particles. In each model, the center pore was set to be the fixed boundary. The expansion during lithiation was simulated by thermal expansion [42,43], the thermal stress *ε* can be given by:

$$\varepsilon =\alpha \left(T-T_{ref}\right)=\alpha \cdot \Delta T$$

where α is the coefficient of thermal expansion, T is the temperature, $T_{ref}$ is the reference temperature, and ΔT is the temperature difference. The model and material parameters are detailed in Table S5. All simulations were performed in COMSOL Multiphysics 5.5 software. Additionally, the deformation of the models was magnified by five times to make the simulation result more visual.

## Data Availability

Data is contained within the article.

## Supplementary Materials

The following are available online at, Figure S1: schematic of the precipitation process, Figure S2: EIS of sCCSi, Figure S3: XPS full spectra of sCCSi, Figure S4: SEM images of CCSi-HT-U, CCSi-HT-M, and CCSi-RT-U, Figure S5: schematics of the possible mechanisms, Figure S6: the distribution of volume strain in nano-silicon particles in CCSi and sCCSi models, Table S1: comparison of the synthesis method of sCCSi with other methods for the synthesis of silicon-based anodes, Table S2: typical pore diameter distribution of sCCSi, Table S3: comparison of the cycle performance of carbon/silicon composites as anode for lithium-ion batteries, Table S4: samples’ names and corresponding synthesis methods, Table S5: the model parameters and material parameters in mechanical simulation.
