# Supplementary material for: Facile Synthesis of Sponge-Like Porous Nano Carbon-Coated Silicon Anode with Tunable Pore Structure for High-Stability Lithium-Ion Batteries
Source: Molecules. 2021 May 27;26(11):3211. doi: 10.3390/molecules26113211 (PMC8199406; doi:10.3390/molecules26113211)
Supplement: Supplementary file 1 [file molecules-26-03211-s001.zip › molecules-1215974-supplementary.pdf]

Supporting Information

Facile Synthesis of Sponge-Like Porous Nano Carbon-Coated Silicon Anode with Tunable Pore Structure for High-Stability Lithium-Ion Batteries

Shugui Song [1], Jingcang Li [1], Anqi Zheng [1], Yongqiang Yang [2,*] and Kuibo Yin [1,*]

[1] SEU-FEI Nano-Pico Center, Key Laboratory of MEMS of Ministry of Education, Southeast University, Nanjing 210096, China; songshugui@seu.edu.cn
[2] National Graphene Products Quality Supervision and Inspection Center (Jiangsu), Jiangsu Province Special Equipment Safety Supervision Inspection Institute, Wuxi 214174, China
* Correspondence: yqyang@wxtjy.com (Y.Y.); yinkuibo@seu.edu.cn (K.Y.)

Keywords: carbon-coated silicon, porous structure, mechanical simulation, lithium-ion battery

Figures and tables

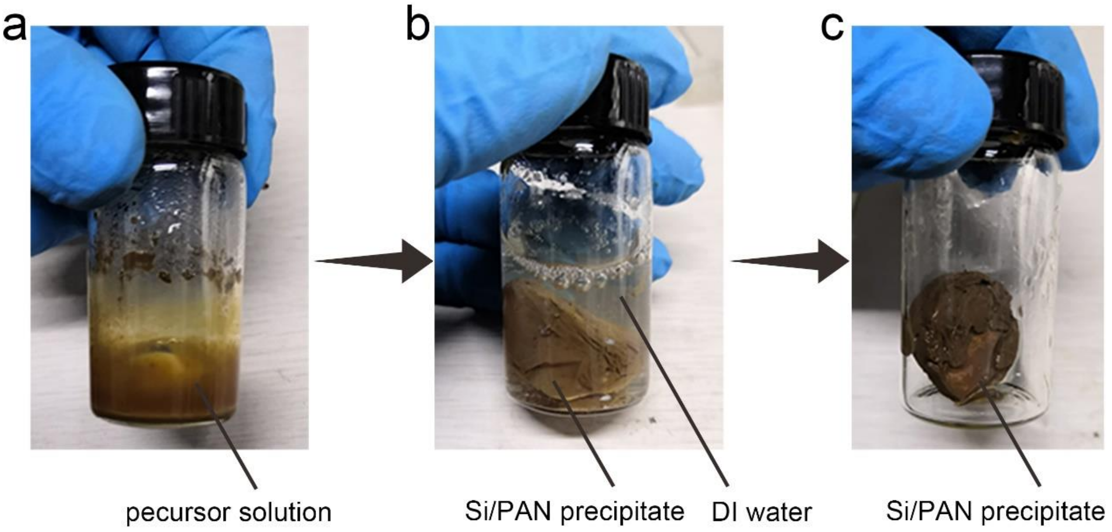

Figure S1. Schematic of the precipitation process. The precursor solution (a) before and (b) after adding DI water. (c) Silicon/polyacrylonitrile (Si/PAN) precipitate.

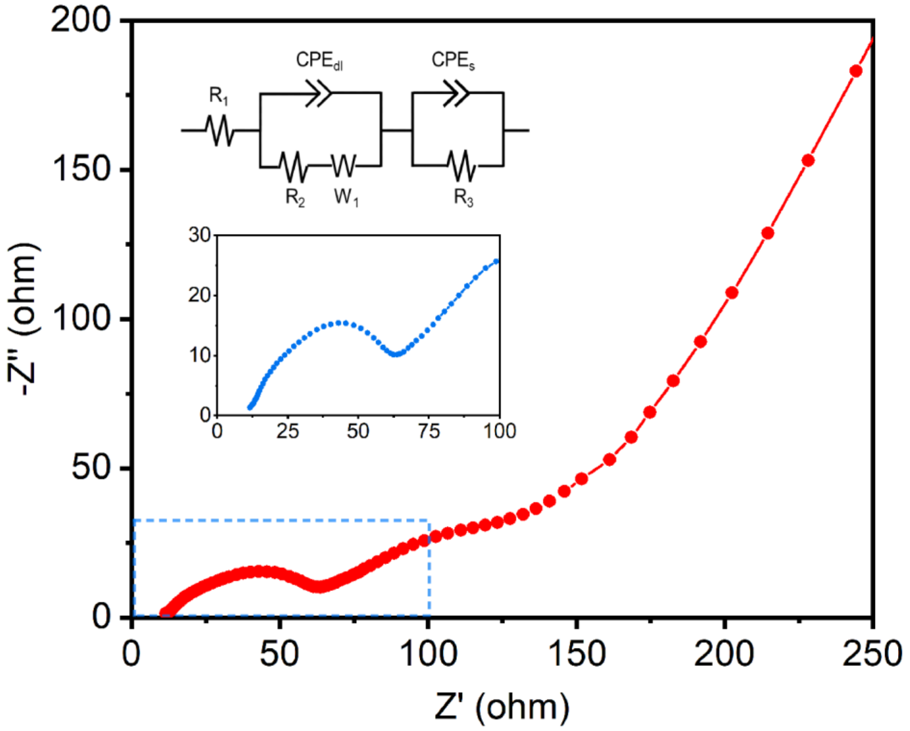

Figure S2. Electrochemical impedance spectroscopy (EIS) of sCCSi.

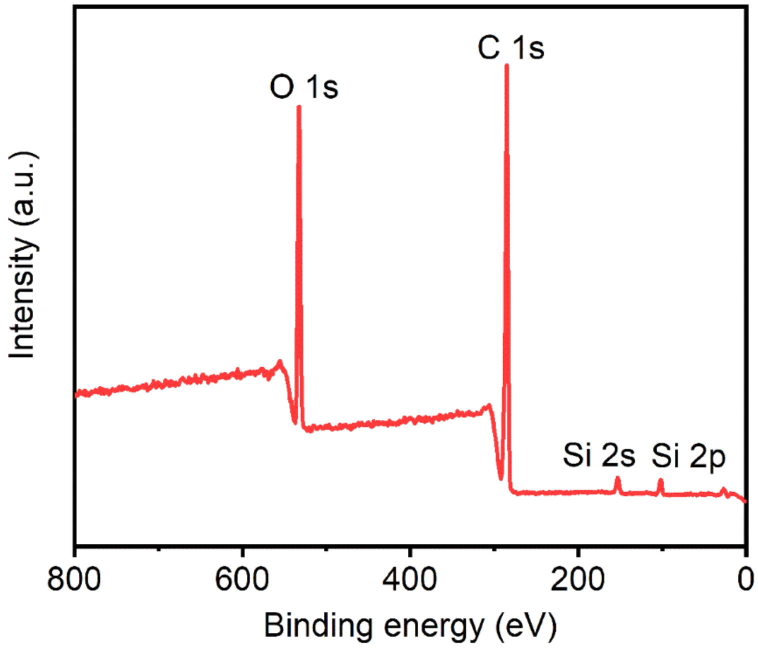

Figure S3. X-ray photoelectron spectroscopy (XPS) full spectra of sCCSi.

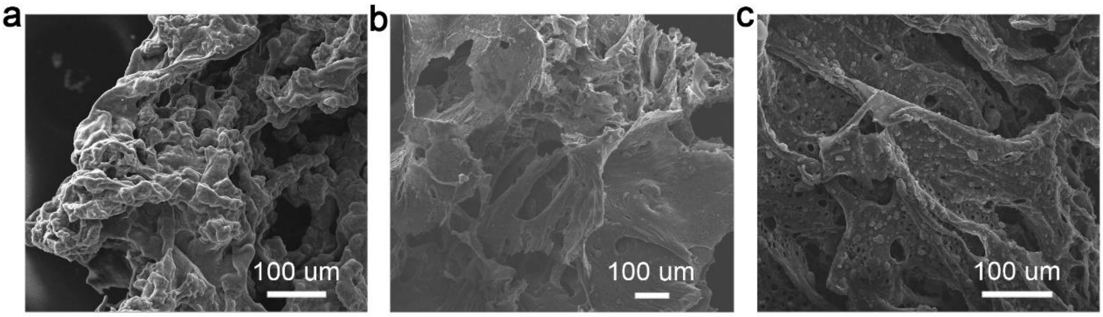

Figure S4. SEM images of (a) CCSi-HT-U, (b) CCSi-HT-M, and (c) CCSi-RT-U.

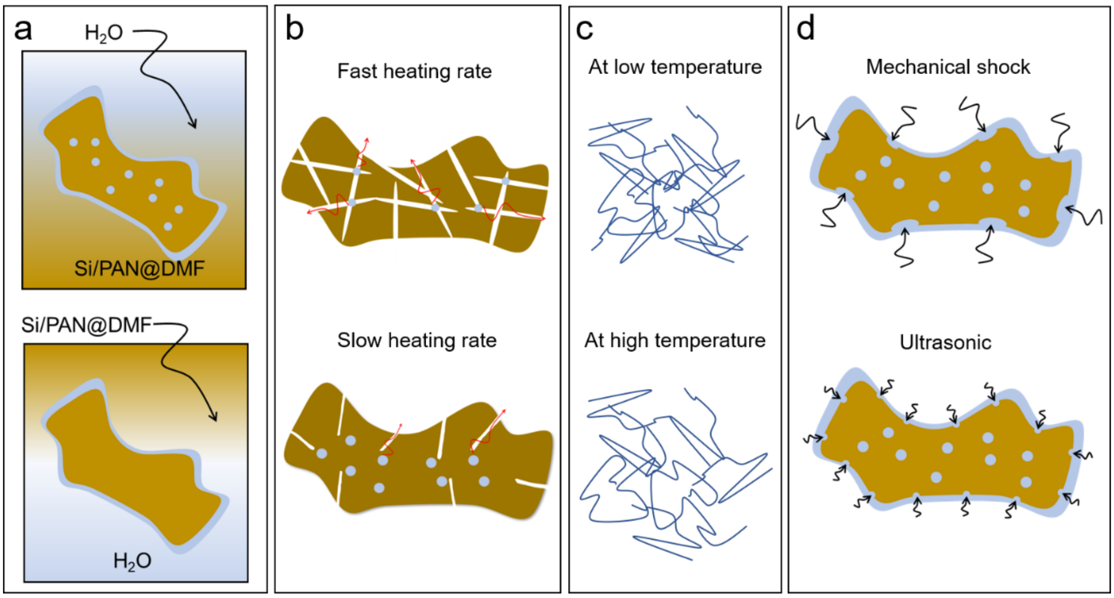

Figure S5. Schematics of the possible mechanisms of (a) water adding strategy, (b) heating rate, (c) stirring temperature, and (d) oscillation method.

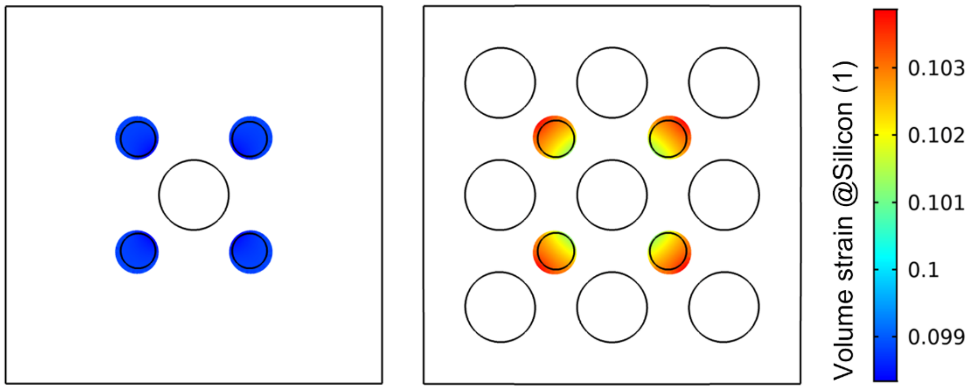

Figure S6. The distribution of volume strain in silicon nanoparticles in CCSi and sCCSi models.

Table S1. Comparison of the synthesis method of sCCSi with other methods for the synthesis of silicon-based anodes.

**Material**	**Involving in etching process**	**Number of preparation steps**	**Cost**	**Ref**
Si/SiOx/C	Yes	5	Medium	(*1*)
Si/C@C	No	5	Medium	(*2*)
Si-C-G-15	No	3	Low	(*3*)
Si/C-ZIF-8/CNFs	No	7	High	(*4*)
Si/C	Yes	5	High	(*5*)
EPSi-mag	Yes	7	High	(*6*)
Si/FLG/C	No	4	High	(*7*)
Si@C@ZIF-67-800N	Yes	9	High	(*8*)
B-Si/CNT@G	Yes	5	High	(*9*)
Si@NC NPs	No	3	Medium	(*10*)
sCCSi	No	4	Low	This work

Note: The ‘Number of preparation steps’ and ‘Cost’ of the synthesis methods are estimated based on the work in the corresponding article. The ‘Number of preparation steps’ is estimated based on important steps such as whether there is a material transfer process and whether heat treatment is performed. The ‘Cost’ is estimated based on the cost of materials, reagents, and equipment used.

Table S2. Typical pore diameter distribution of sCCSi.

	**Number of samples**	**Mean (nm)**	**Standard deviation (nm)**	**Minimum (nm)**	**Median (nm)**	**Maximum (nm)**
Porous structure 1	100	191	75	67	183	434
Porous structure 2	100	2409	929	471	2252	5818
Porous structure 3	100	8342	1924	4186	8136	13889

Table S3. Comparison of the cycle performance of carbon/silicon composites as anode for lithium-ion batteries.

**Material**	**Silicon content**	**Capacity ($mAh\ g^{-1}$)**	**Capacity retention @cycles**	**Current density ($mA\ g^{-1}$)**	**Ref**
Si/SiOx/C	10%	780	65% @100	400* (0.5 C)	(*1*)
Si/C@C	27.4%	602	80% @100	1000	(*2*)
Si-C-G-15	15%	712	80% @100	130	(*3*)
Si/C-ZIF-8/CNFs	54.5%	945.5	64% @150	200	(*4*)
Si/C	21.8%	997	71% @150	200	(*5*)
EPSi-mag	50%	1000	78% @150	300*	(*6*)
Si/FLG/C	51.9%	757	62% @300	400* (0.5 C)	(*7*)
Si@C@ZIF-67-800N	7.6%*	852	74%* @300	1000	(*8*)
B-Si/CNT@G	26.7%*	180#	82.5% @300	180	(*9*)
Si@NC NPs	11.8%	967.1	87.3% @300	800* (1 C)	(*10*)
sCCSi-15	16.7%	726.7	70.6% @300	500	This work
sCCSi-12	33.3%	1173.1	70.2% @300	500	This work

Notes: (1) Data with '*' are estimated values. (2) Data with '#' are from full cell LIBs.

Table S4. Samples' names and corresponding synthesis methods.

Method 1	Method 2	Name
Add precursor solution to DI water	/	sCCSi-w
Vacuum drying	/	CCSi-v
Oven drying	/	CCSi-o
High temperature (80 ℃)	Ultrasonic shake	CCSi-HT-U
High temperature (80 ℃)	Mechanical shake	CCSi-HT-M
Room temperature (25 ℃)	Ultrasonic shake	CCSi-RT-U

Table S5. The model parameters and material parameters in mechanical simulation.

**Parameter**	**Value**	**Unit**	**Definition**
l	270	nm	Length of sCCSi model
$R_{silicon}$	25	nm	Silicon diameter in sCCSi model
R_{pore}	50	nm	Pore diameter in sCCSi model
T_{ref}	293.15	K	Reference temperature
T	1000	K	Thermal expansion temperature
α_{carbon}	$1 \cdot 10^{-7}$	K^{-1}	Thermal expansion coefficient of carbon
ρ_{carbon}	1.7	g cm^{-3}	Density of carbon
E_{carbon}	200	GPa	Young's modulus of carbon
ν_{carbon}	0.3	1	Poisson's ratio of carbon
$\alpha_{silicon}$	$9 \cdot 10^{-5}$	K^{-1}	Thermal expansion coefficient of silicon
$\rho_{silicon}$	2.33	g cm^{-3}	Density of silicon
$E_{silicon}$	140	GPa	Young's modulus of silicon
$\nu_{silicon}$	0.265	1	Poisson's ratio of silicon

REFERENCES

1. C.-C. Hsieh, Y.-G. Lin, C.-L. Chiang, W.-R. Liu, Carbon-coated porous Si/C composite anode materials via two-step etching/coating processes for lithium-ion batteries. *Ceram. Int.* **46**, 26598-26607 (2020).
2. Y. Li *et al.*, Sandwich structure of carbon-coated silicon/carbon nanofiber anodes for lithium-ion batteries. *Ceram. Int.* **45**, 16195-16201 (2019).
3. S. Y. Kim *et al.*, Facile Synthesis of Carbon-Coated Silicon/Graphite Spherical Composites for High-Performance Lithium-Ion Batteries. *ACS Appl. Mater. Interfaces* **8**, 12109-12117 (2016).
4. Y. Zeng *et al.*, N-doped porous carbon nanofibers sheathed pumpkin-like Si/C composites as free-standing anodes for lithium-ion batteries. *Journal of Energy Chemistry* **54**, 727-735 (2021).
5. H. Zhang *et al.*, Electrospun core–shell silicon/carbon fibers with an internal honeycomb-like conductive carbon framework as an anode for lithium ion batteries. *Journal of Materials Chemistry A* **3**, 7112-7120 (2015).
6. X. Li *et al.*, A stable nanoporous silicon anode prepared by modified magnesiothermic reactions. *Nano Energy* **20**, 68-75 (2016).
7. C.-C. Hsieh, W.-R. Liu, Carbon-coated Si particles binding with few-layered graphene via a liquid exfoliation process as potential anode materials for lithium-ion batteries. *Surf. Coat. Technol.* **387**, 125553 (2020).
8. N. Liu *et al.*, Multi-core yolk-shell like mesoporous double carbon-coated silicon nanoparticles as anode materials for lithium-ion batteries. *Energy Storage Mater.* **18**, 165-173 (2019).
9. P. Li, J.-Y. Hwang, Y.-K. Sun, Nano/Microstructured Silicon–Graphite Composite Anode for High-Energy-Density Li-Ion Battery. *ACS Nano* **13**, 2624-2633 (2019).
10. H. S. Choi *et al.*, Enhanced cycle stability of silicon nanoparticles coated with nitrogen-doped carbon layer for lithium-ion battery anode. *Current Applied Physics* **17**, 1087-1093 (2017).
